# In-vitro dehydration kinetics coefficient of Kalifilcon A and other contact lens materials

**DOI:** 10.1038/s41598-024-55937-2

**Published:** 2024-04-03

**Authors:** Erika Ponzini, Francesco Maspero, Anna Galli, Silvia Tavazzi

**Affiliations:** 1 Materials Science Department, University of Milano Bicocca, Via R. Cozzi 55, 20125 Milan, Italy; 2 COMiB Research Center, University of Milano Bicocca, Via R. Cozzi 55, 20125 Milan, Italy

**Keywords:** Gels and hydrogels, Polymers, Characterization and analytical techniques

## Abstract

In contact lens (CL) wear, dehydration needs to be tailored to avoid dryness and related symptoms. In this view, this work aims to assess and compare the in-vitro dehydration kinetics of five CL materials, including the newly developed Kalifilcon A CL. At 36 °C and 60% relative humidity, the in-vitro dehydration kinetics of the different CLs were compared using a gravimetric method. CLs were analyzed either after a rinse of a few seconds in preservative-free saline solution or after a 24-h incubation in the same solution. A model based on the Fick diffusion equation was employed to deduce a water kinetics coefficient, providing insights into water diffusion within the polymeric matrix. The study reveals that all materials exhibit a non-Fickian dehydration behavior, with significant differences in dehydration kinetics coefficients and dehydration rate slopes. Etafilcon A and Omafilcon A, both hydrogel CLs, exhibit a similar behavior, different compared to the pattern shown by Senofilcon A and Delefilcon A, silicone-hydrogel CLs. Notably, Kalifilcon A, despite being a silicone-hydrogel, displays a hydration behavior reminiscent of hydrogel CLs.

## Introduction

The interaction with water is a crucial property of soft contact lenses (CLs) influencing not only the lens material, its geometry, and the tear film, but also the corneal epithelium on the ocular surface^1^. The tendency of CLs to dehydrate plays a significant role in complaints of dryness and other related symptoms during wear^1–4^, often associated with increased ocular inflammation, a focal point of interest due to the intrinsic inflammatory nature of CL wear^5^. Dehydration initiates when a CL is placed on the eye and continues during wear, influenced by various factors such as material composition, physical properties, modifications during wear, CL thickness, environmental conditions, tear composition, and blink rate^1,6–8^.

Recently, a novel daily disposable silicone-hydrogel (SiHy) material named Kalifilcon A was developed. According to the manufacturer’s declaration, the characteristics of Kalifilcon A are designed to stabilize the tear film, maintain ocular surface homeostasis, and retain hydration, addressing the needs of CL wearers experiencing discomfort and dry eye symptoms. Composed of a hydrophilic siloxane copolymer of 2-hydroxyethyl methacrylate (HEMA) and N-vinylpyrrolidone (NVP), Kalifilcon A undergoes a two-phase polymerization process: first, long- and short-chain silicone polymers create the silicone backbone in which dimethylacrylamide (DMA), a hydrophilic compound, is integrated; second, polyvinylpyrrolidone (PVP) is permanently grown throughout the CL matrix. A similar process has been reported also for Samfilcon A^9^, a polymer from the same manufacturer designed for monthly replacement CLs. Compared to other SiHy CLs, Kalifilcon A exhibits a relatively high equilibrium water content (EWC), similar to typical hydrogel (Hy) values^10^. Its packaging solution includes moisturizing components (poloxamine and poloxamer) and osmoprotectants (erythritol and glycerin). Reindel et al.^11^ conducted in-vivo performance evaluations of Kalifilcon A CLs, focusing on habitual planned-replacement SiHy CL wearers involved in regular physical activity (PA) and those who were not (NPA). The study aimed to explore the impact of physical exercise on both body hydration status and eye blink rate, factors that could lead to hyperosmotic tears. The authors reported positive outcomes, indicating that Kalifilcon A CLs performed well in terms of comfort and vision ratings for both PA and NPA subjects. Notably, subjects engaged in physical activity provided more favorable ratings associated with vision and comfort. Similarly, a following study conducted clinical evaluations of Kalifilcon A CLs in subjects with and without dryness symptoms, providing further insights into their clinical performance^12^. Also in this case, both subgroups exhibited substantial concordance with all comfort and vision attribute statements. Notably, the dryness subgroup indicated elevated levels of agreement with most comfort-related statements.

The positive outcomes observed in comfort and vision ratings highlight the potential benefits of Kalifilcon A in addressing the discomfort and dry eye symptoms experienced by CL wearers. These observations inspired the current in-vitro investigation, which seeks to determine whether the unique properties of Kalifilcon A, reported in real-world scenarios, manifest also in its dehydration characteristics concerning water retention and resistance to dehydration. In a controlled environment, in-vitro analyses can provide insights into the effects of the chemical and physical properties of the CL polymeric matrix on its dehydration. Refractometry and gravimetric methods are commonly employed for this purpose. While refractometry relies on principles such as totally internal reflection, gravimetric methods with digital microbalances are considered more accurate^1,13–15^. This work aims to assess and compare the in-vitro dehydration kinetics of five CL materials, including the newly developed Kalifilcon A CL. Employing a model based on the Fick's diffusion equation, a quantitative parameter, the water kinetics coefficient, is proposed to characterize water diffusion in the polymeric matrix and compare different materials. The relationship between this parameter and the shapes of dehydration curves reported in the literature is discussed in detail.

## Materials and methods

### Materials

CLs with the same back vertex power (− 0.50 diopters) of five different materials were studied (Table 1).

**Table 1:** List of the investigated CL materials and their parameters as provided by the manufacturers, except the packaging solution composition in the case of Omafilcon A.

Material	Omafilcon A (Hy)	Etafilcon A (Hy)	Senofilcon A (SiHy)	Delefilcon A (SiHy)	Kalifilcon A (SiHy)
Brand	Proclear1 Day	1Day Acuvue Moist	Acuvue Oasys	Dailies Total1	Infuse/Ultra One Day
Manufacturer	CooperVision	Johnson&Johnson	Johnson&Johnson	Alcon	Bausch + Lomb
Type of CL	Hy	Hy	SiHy	SiHy	SiHy
FDA group (subgroup)	II	IV	V (C)	V (C)	V (B)
Oxygen transmissibility (Dk/t)	28	33	121	156	134
Modulus (MPa)	0.49	0.30	0.72	0.7	0.5
Manufacturing method	Cast molded	Cast molded	Cast molded	LightStream	Cast molded
EWC (%)	60	58	38	33 (outer layer > 80)	55
t_c_ (µm)	68	84	70	90	80
Principal monomers	HEMA, MPC^16,17^	HEMA, MAA, PVP^17^	HEMA, DMA, PDMS, siloxane macromer, TEGDMA, PVP^16^	Si-Hy (core), Non-silicone hydrophilic polymer (surface)	DMA, HEMA, siloxane macromer, PVP
Surface treatment	None	None (PVP as internal wetting agent, LACREON)^18^	None (PVP as internal wetting agent, HydraLuxe)^18^	Water gradient technology (LightStream) ^19^	None (PVP as internal wetting agent, Advanced MoistureSeal)
Packaging solution	Buffered saline (plus surface-active agents)^20^	Borate buffered saline with povidone	Buffered saline with methyl ether cellulose	Buffered saline solution with approximately 0.3% of polymeric wetting agents consisting of copolymers of polyamidoamine and poly(acrylamide-acrylic acid)	Phosphate buffered saline with potassium chloride, poloxamine 1107, poloxamer 181, glycerin, and erythritol (ComfortFeel)

All investigated CL materials are daily disposable. EWC is the equilibrium water content, t_c_ is the central thickness of monofocal CLs of power equal to − 3.00 diopters, Dk/t is the oxygen transmissibility of monofocal CLs of power equal to − 3.00 diopters (10^–9^ mL O_2_/(cm^2^ s mmHg)). The classification into groups and subgroups is reported in accordance with ISO 18369-1:2017. *DMA* dimethylacrylamide, *FDA* Food and Drug Administration, *HEMA* poly-2-hydroxyethyl methacrylate, *Hy* Hydrogel CLs, *MAA* methacrylic acid, *MPC* 2-methacryloxyethyl phosphorylcholine, *NVP* N-vinyl pyrrolidone, *PDMS* polydimethylsiloxane, *PVP* poly(vinyl pyrrolidone), *SiHy* Silicone-hydrogel, *TEGDMA* tri ethylene glycol dimethacrylate.

Except for the composition of the blister solution of Omafilcon A, the other parameters reported in Table 1 are provided by the manufacturers. The additional information on the presence of surface-active ingredients in Omafilcon A packaging was reported by Lin and Svitova, who measured the surface tension of the packaging solution of several commercially available CLs^20^.

For each material, three CLs were rinsed for a few seconds in saline solution and three CLs were incubated for 24 h. The solution employed for rinsing or incubating the CLs was a preservative-free saline solution (Salisin, Schalcon, Rome, Italy).

### Dehydration

First, to measure for each material the mass of the dehydrated samples (M_deh_), fully dehydrated CLs were obtained by lyophilization with a freeze dryer (Scanvac, Analytical control De Mori, Milan, Italy) and their M_deh_ was measured using an analytical balance (Entris, Sartorius, Göttingen, Germany).

To investigate the in-vitro dehydration kinetics, CLs were dehydrated under controlled environmental conditions (temperature 34 °C, relative humidity 60%)^21^ on a dynamic vapor sorption analyzer (Quantachrome Instruments Aquadyne DVS). The device has a capacity of 5 g, weighing to 0.1 μg, and maintains tightly controlled environmental conditions of relative humidity (± 0.1%) and temperature (± 0.2 °C) in an enclosed weighing chamber. The mass was recorded every 30 s for a period of 120 min (at most). The environmental conditions were kept constant throughout each experiment^22^. The balance pan was adapted placing a plastic polydimethylsiloxane spherical holder (radius of curvature of 7.7 mm) on it. After the stabilization of the instrument, the CLs were placed on the holder and the dehydration profiles were measured by weighing the CLs as time varies and were carried out starting from CLs with a water content higher than the EWC until the complete lens dehydration.

Although the CL surface may be blotted before analyses^23^, the blotting procedure was here avoided as it could compromise the starting mass value and the repeatability between measurements^18^.

A protocol was identified to ensure consistency and improve reproducibility. Unworn CLs were either taken from their packaging blister and quickly rinsed (approximately 2 s) in 5 mL of saline solution or taken from the blister and incubated for 24 h in 5 mL of saline solution. Incubation in saline solution prior to measurements is commonly employed to remove most of the wetting agents from the packaging solutions as well as other components released from the CL^19,24^.

## Results and discussion

Table 2 shows the mass values of fully dehydrated CLs after lyophilization.

**Table 2:** Mass values of fully dehydrated CLs after lyophilization.

Material	M_deh_ (mg)
Omafilcon A	14.1 ± 0.1
Etafilcon A	15.4 ± 0.1
Senofilcon A	18.2 ± 0.3
Delefilcon A	19.9 ± 0.3
Kalifilcon A	13.1 ± 0.4

Values (mean ± standard deviation) of the mass M_deh_ of completely dehydrated CLs of the five investigated materials.

**Figure 1:**
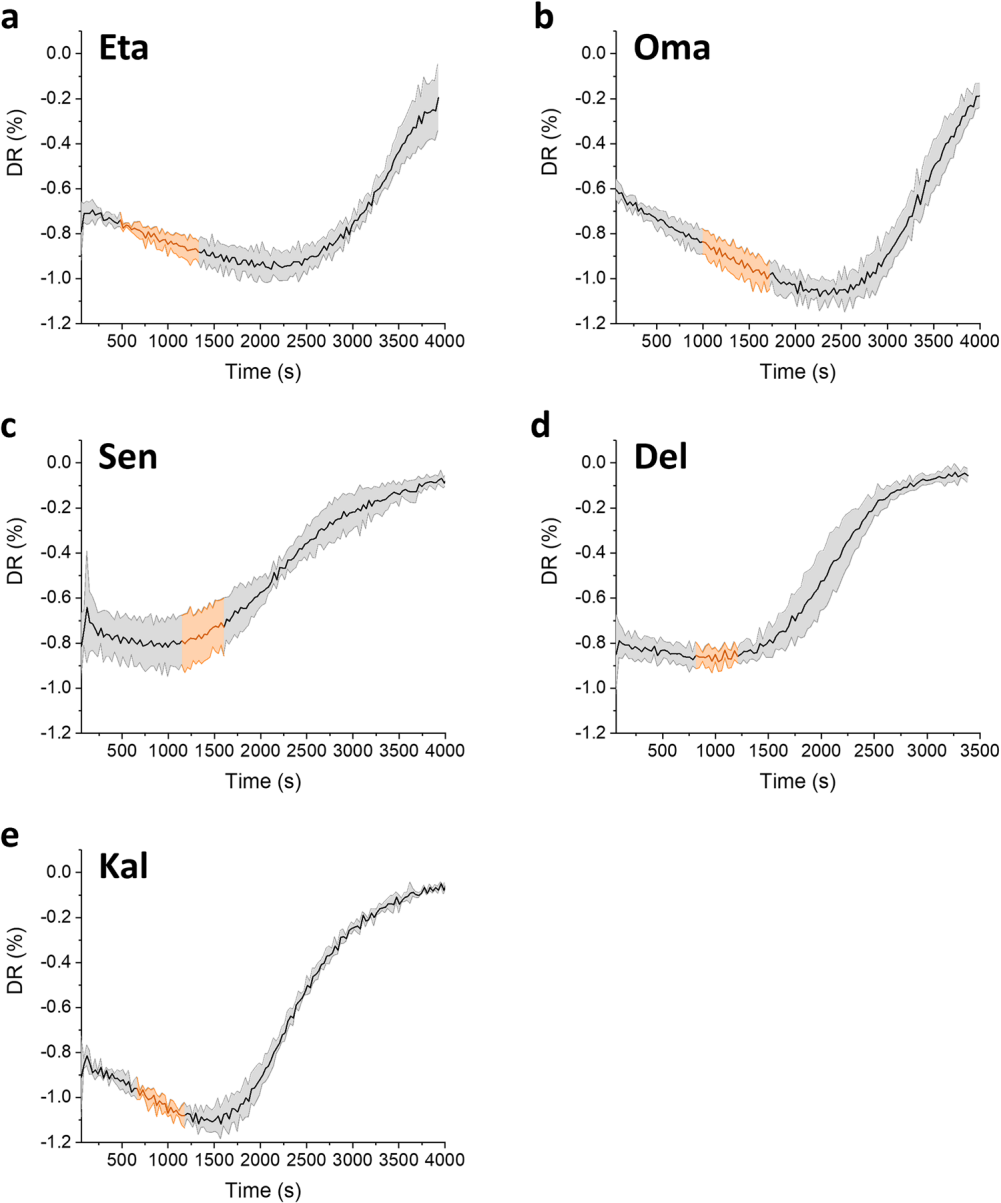
Curves of DR for each CL material analyzed in this study. The line represents the mean DR value calculated over four independent experiments, whereas the coloured area represents the standard deviation. DR is defined as $\left[\frac{{{M}_{t(n)}-M}_{t\left(n-1\right)}}{{M}_{t(n)}}\right]$, where *M*_*t*(*n*)_ is the sample weight at time *n* and *M*_*t(n−1)*_ the sample weight at time (*n* – 1) with intervals of 30 s. The orange region highlights the DR profile from the mass at the EWC (M_t100%_) to a mass equivalent to a 35% of water loss (M_t65%_). CL materials are abbreviated as follows: Oma, Omafilcon A; Eta, Etafilcon A; Sen, Senofilcon A; Del, Delefilcon A; Kal, Kalifilcon A.

Figure 1 displays the dehydration rate (DR) mean values, obtained as a function of time on the five different CL materials, DR being defined as:

1 $$DR=\left[\frac{{{M}_{t(n)}-M}_{t\left(n-1\right)}}{{M}_{t(n)}}\right]$$

where *M*_*t*(*n*)_ is the sample weight at time *n* and *M*_*t(n−1)*_ the sample weight at time (*n* − 1) with intervals of 30 s.

The orange region in each panel highlights the dehydration profile from the mass at the EWC (M_t100%_) to a mass equivalent to a 35% of water loss (M_t65%_). For each material, the curves showed similar trends after a brief rinse or after a 24-h in saline solution, as evidenced by the relatively low standard deviations in Fig. 1 calculated starting from all the measurements carried out both after a short rinse and after a long incubation. Therefore, the two conditions were not considered as variables in all the following analyses. In most cases, the dehydration curves of the same material could be reasonably superimposed, with Kalifilcon A CLs showing the lowest standard deviation, i.e., the highest repeatability.

The three-phase pattern described in the literature by González-Méijome et al.^1^ can be observed in Fig. 1. These authors performed the analyses at 22.4 °C and 49.1% of relative humidity after blotting the CLs on filter paper. Although the analyses of the present work were conducted on different materials, at 34 °C and 60% of relative humidity with no blotting, a similar three-phase behavior was observed. Phase 1 (P1) ranges from the beginning of the experiment until the minimum DR. The length of P1 depends on the hydration of the CL at the beginning (t_0_) of the analysis, which can be higher than the EWC (this is always the case, for example, in Fig. 1). Therefore, a higher hydration at t_0_ is expected to lead to a longer P1, unless the starting condition is fixed a priori (for example making sure that the initial hydration corresponds to M_t100%_). After reaching a minimum DR, each CL shows a rapid and progressive increase in the DR, which represents phase 2 (P2). Then, the CL approaches a DR value equal to zero, and this last part of the profile was defined as third phase (P3) although no distinct change can be defined between the phases^1^.

Since CLs worn in vivo are expected to have equal or slightly less hydration than the EWC^8,25,26^, a first aspect that is shown in Fig. 1 is the range of the DR profile from the mass at the EWC (M_t100%_) to a mass equivalent to a 35% of water loss (M_t65%_). This range (M_t100%_ − M_t65%_) does not correspond to the same phase for all materials. For both hydrogels (Hys, Omafilcon A and Etafilcon A), the range falls within P1 and it is far from the DR minimum. On the contrary, for Senofilcon A and Delefilcon A, both silicone-hydrogels (SiHys), the range is close to or even includes the DR minimum. Interestingly, in the case of Kalifilcon A, which is a SiHy with a relatively high EWC, the range falls clearly within P1. An effect of the material composition on the dehydration profile was discussed by González-Méijome et al., who highlighted that P1 is characteristic for Hy CLs and that there is a positive correlation between its duration and the EWC. These authors also underlined that this is not always true for SiHy CLs, in particular for siloxane-rich materials, which may even lack a P1. González-Méijome et al. adopted the blotting procedure which is expected to eliminate the excess of water on the surface. Therefore, their starting point is expected to reasonably correspond to the condition here defined as M_t100%_ (left end of the orange line in Fig. 1). Based on these considerations, excluding Kalifilcon A, the results obtained in this work are in good agreement with what reported by González-Méijome et al. because, starting from M_t100%_, the two Hys in Fig. 1 show a relatively long P1, while the two SiHys (excluding Kalifilcon A) substantially do not show any P1. Kalifilcon A is a high-EWC SiHy, but shows the P1 phase and it appears, as already mentioned, to behave as Hy CLs.

A way to define mathematically the position of the range of interest, i.e., from M_t100%_ to M_t65%_, with respect to the minimum DR is to calculate the slope of the DR linear fitting in the M_t100%_ − M_t65%_ range: negative slopes indicate that the 35% of the initial water content is lost during P1, positive values indicate that the loss takes place during P2, whereas values close to zero suggest that the evaporation happens during the transition from one phase to the other. When comparing the different materials, negative slope values were measured for the two Hys Omafilcon A and Etafilcon A, as well as for Kalifilcon A, a SiHy CL, despite the siloxane-rich hydrophobic component of the latter. A similar observation was reported also in the work by González-Méijome et al.^1^, where no significant difference was found between the dehydration behavior observed when comparing a SiHy CL (38%-EWC Senofilcon A) and conventional Hy CLs with similar EWC (36%-EWC Balafilcon A and 38.6%-EWC Polymacon). The authors concluded that the EWC has a pivotal role in determining the dehydration characteristics of CLs, which is a consistent observation across different studies^26–28^. Nonetheless, without considering the specific monomeric composition of CLs, which is not always available, a potential contribution of other intrinsic properties of the material itself cannot be excluded. Positive slope values were measured for Delefilcon A and Senofilcon A SiHy CLs. Interestingly, Senofilcon A displayed the highest value and has the lowest EWC (38%) compared to all the other CLs under investigation. Delefilcon A (bulk EWC 33%) shows a slope more similar to Senofilcon A SiHy CL than to high-EWC CLs, despite its Hy coating^14^. Indeed, the surface layer is thin and dehydrates so rapidly that the CL mainly behaves like its low-EWC bulk, as suggested by other experimental evidences present in the literature^14,15,29^. For example, refractometry studies characterized the surface layer of water-gradient CLs, evidencing an initial refractive index of 1.34, compatible with the high EWC of the outer hydrogel layer of Delefilcon A^15^. However, other authors reported a refractive index of 1.43^30^. Considering that the surface layer with 80% hydration should have a refractive index very similar to that of water, the latter value could have been overestimated, perhaps due to the rapid dehydration.

**Figure 2:**
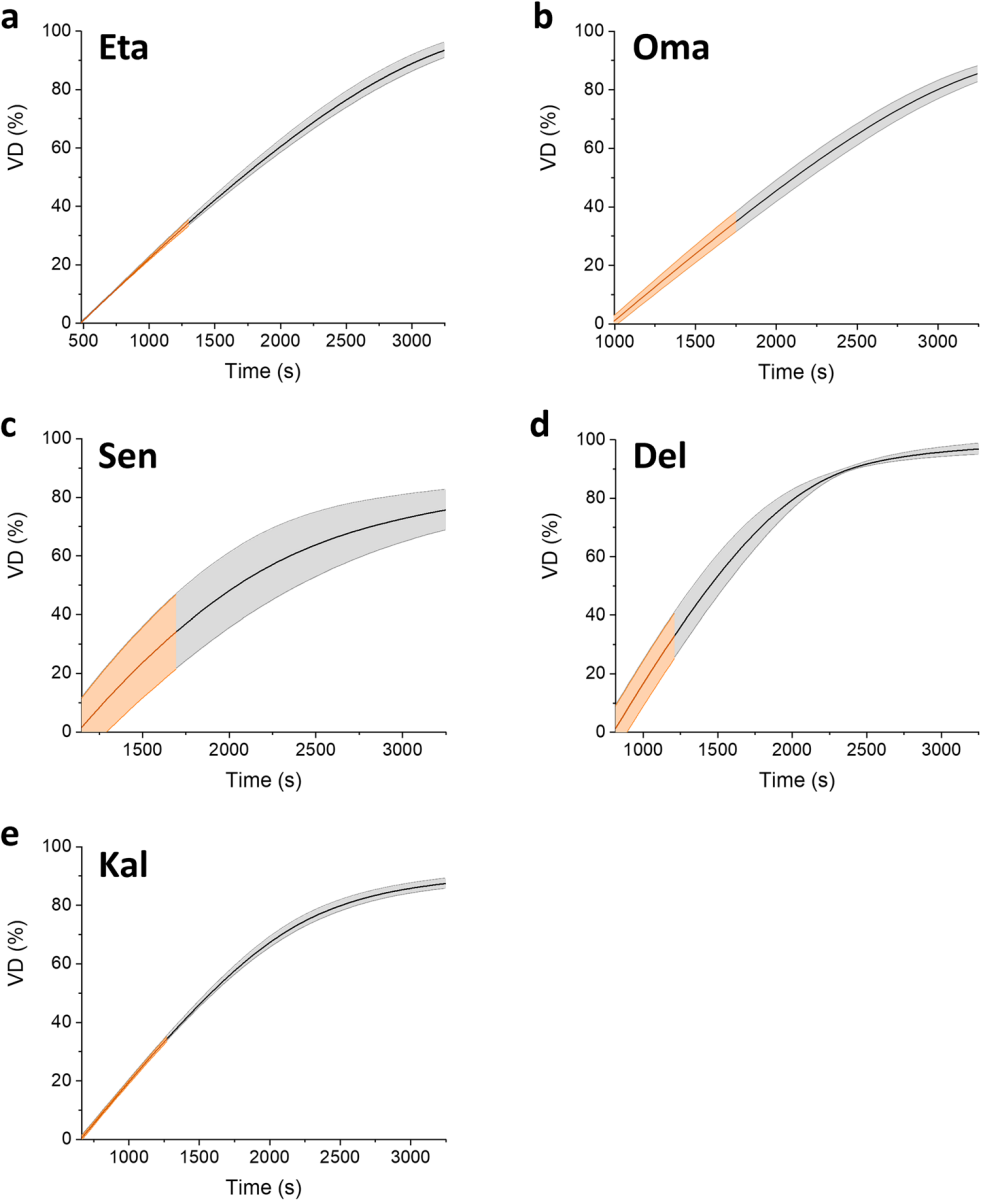
Curves of VD for each CL material analyzed in this study. The line represents the mean VD value calculated over independent experiments, whereas the colored area represents the standard deviation. The orange region highlights the VD profile from the mass at the EWC (M_t100%_) to a mass equivalent to a 35% of water loss (M_t65%_). CL materials are abbreviated as follows: Oma, Omafilcon A; Eta, Etafilcon A; Sen, Senofilcon A; Del, Delefilcon A; Kal, Kalifilcon A.

The analysis of the DR curves is one of the approaches reported in the literature to describe the in-vitro dehydration of CLs. A previous study correlated the evaporative water loss with water self-diffusion^31^. The authors concluded that the change in DR during time is an effect of diffusion and high-EWC CLs are able to maintain a high rate of diffusion of water to the evaporative surface due to a higher relative amount of mobile water molecules^31^. Another parameter, described by González-Méijome et al. with the name of valid dehydration (VD)^1^, is displayed in Fig. 2 as obtained by Eq. (2):

2 $$VD=\left[\frac{{{M}_{t(n)}-M}_{t100\%}}{{{M}_{deh} - M}_{t100\%)}}\right]$$

where *M*_*t100%*_ is the sample weight in the hydration condition corresponding to the EWC, *M*_*t*(*n*)_ is the sample weight at time *n* with intervals of 30 s, M_deh_ is the final sample weight (dried condition).

The VD curve allows to deduce a quantitative parameter that directly describes the dehydration kinetics of the lens. This parameter is proposed as an indicator to characterize the process of water diffusion in the polymeric matrix and to compare different materials. Indeed, as reported in Eq. (3), VD defined by Eq. (2) corresponds to the cumulative amount of water loss (m_t_) at time t_(n)_ in relation to the total amount of water that can be removed from the polymer (i.e., the total mass m_∞_ of water at the EWC). This ratio can be employed to further investigate water diffusion mechanisms through the Fick’s model. Fick’s first law predicts the mass flux in a gradient of concentration in space, whereas Fick’s second law describes how diffusion affects concentration along time. Considering a one-dimensional and isothermal water release in a polymer slab or tablet, the solution of Fick’s second equation is^32^:

3 $$VD= \frac{{m}_{t}}{{m}_{\infty }}=k{t}^{d}$$

where *k* is a constant and *d* describes the water diffusion^32^. Through this equation and the VD curves measured on the different samples, the d and k coefficients of the five materials under investigation have been deduced in order to describe the dehydration process in the interval VD < 0.35 (Table 3). This interval corresponds to the range of water content of the CL from its EWC to the 35% of water loss (the range indicated in orange in Figs. 1 and 2). In case of pure Fickian diffusion, d is expected to assume the limit value of 0.50 for slabs and to be comprised between 0.43 and 0.50 for tablets, depending on the ratio between diameter and thickness^32^.

**Table 3:** d and k coefficients for each material.

Material	d	k	m_∞_ (mg)
Omafilcon A	0.990 ± 0.007	0.00047 ± 0.00003	21.2 ± 0.2
Etafilcon A	0.989 ± 0.014	0.00047 ± 0.00005	21.3 ± 0.1
Senofilcon A	0.969 ± 0.015	0.00090 ± 0.00007	11.2 ± 0.2
Delefilcon A	0.974 ± 0.017	0.00094 ± 0.00013	9.8 ± 0.2
Kalifilcon A	0.991 ± 0.007	0.00065 ± 0.00003	16.0 ± 0.5

Mean ± standard deviation of the measured values is reported, together with m_∞_ deduced from the EWC and M_deh_ values described in Tables 1 and 2 ((M_deh_/m_∞_) = (1 − EWC)/EWC).

Despite the limitations of a one-dimensional model for a slab or a tablet, the d values in Table 3 suggest that all materials dehydrate following a non-Fickian model, being always very close to 1^32^. According to this observation, k can be seen as the angular coefficient of a line as a function of time t with approximated equation VD = k·t [assuming d = 1 in Eq. (3)]. In other words, in first approximation k is inversely proportional to the time that is needed by the CL to lose the 35% of its water content. In this view, the materials with higher k values, i.e., Senofilcon A and Delefilcon A, need less time to lose the 35% of their water, but simply because they contain much less water in the initial condition M_t100%_ (EWC). Interestingly, Kalifilcon A presents an intermediate behavior, but, also in this case, the conclusion is straightforward because it contains an intermediate mass of water in the M_t100%_ condition (Table 3).

**Figure 3:**
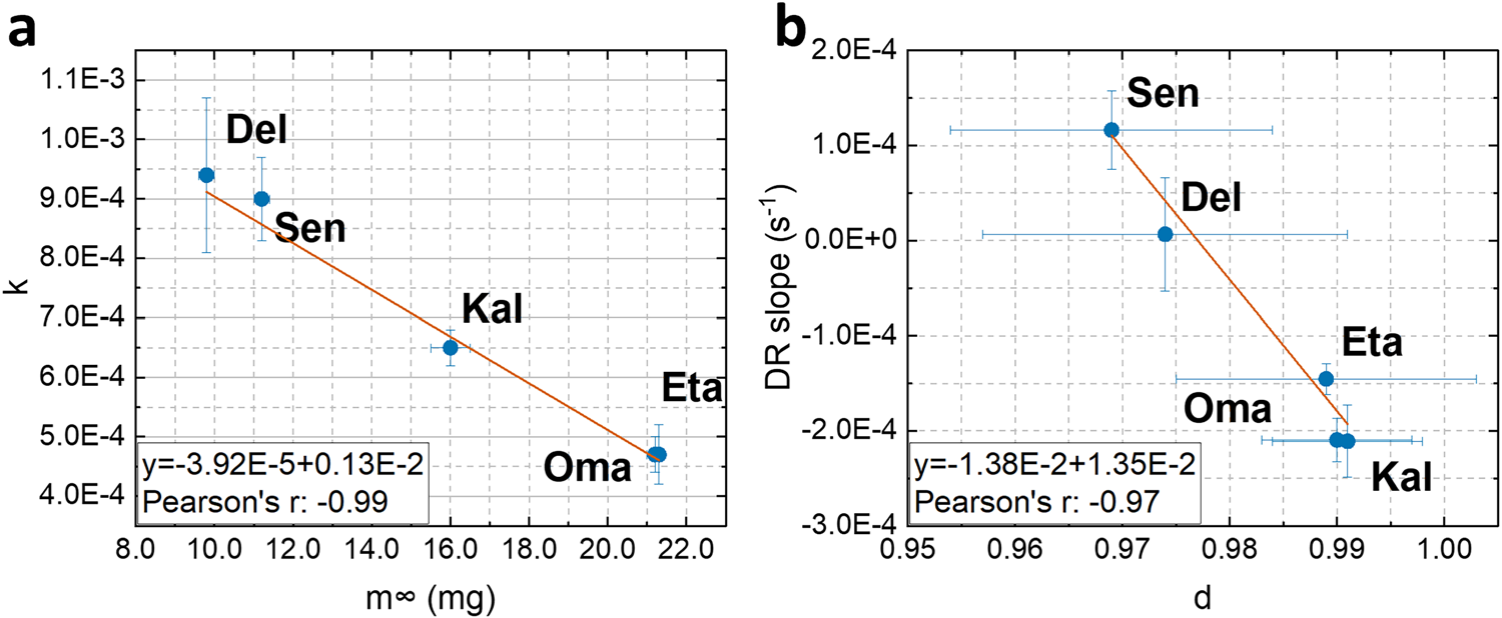
Relationship between dehydration parameters. Relationship between k and m_∞_ (**a**) and between DR slope and d (**b**).

Statistical analysis could be applied to compare the k parameters of different materials. However, this comparison was not performed because k is strongly dependent on the EWC of the material under investigation. Significant differences are expected between the materials, but this result is trivial when considering the well-known and very different values of EWC. Considering the meaning of each variable, it is not surprising to find a strong relationship (Pearson’s correlation coefficient = − 0.99) between the k values and the total amount m_∞_ of water that is available in the polymer (Fig. 3a).

If the correlation between k and m_∞_ was predictable, Fig. 3b shows that a strong correlation is also found between the DR slope in the range M_t100%_ − M_t65%_ and the parameter d (Pearson correlation coefficient equal to − 0.97). Despite the similar behavior in terms of non-Fickian diffusion mechanism (d values are always very close to 1), some differences between the d values of the different materials were found by the Mann–Whitney *U* test, as shown in Table 4, where p-values lower than the threshold of statistical significance (taken equal to 0.10) are shown in bold. The Mann–Whitney *U* test was also applied to the M_t100%_-M_t65%_ DR slope values (Table 4). Considering the strong correlation between d and this slope (Fig. 3), both values provide information about water diffusion, and it is not surprising that similar statistical differences are highlighted. As expected on the basis of Fig. 1, both d values and DR slope values evidence very clearly the difference between, on the one hand, the two Hy (Omafilcon A and Etafilcon A) and, on the other, the two SiHy materials (Senofilcon A and Delefilcon A), as well as the Hy-like behavior of the Kalifilcon A SiHy CL.

**Table 4:** Statistical differences among materials according to d and DR slope values.

DR slope	Oma A	Eta A	Sen A	Del A	Kal A
d					
Oma A		0.7015	**0.0107**	**0.0230**	0.5203
Eta A	0.4761		**0.0107**	**0.0148**	**0.0741**
Sen A	**0.0029**	**0.0336**		0.3913	**0.0142**
Del A	**0.0526**	**0.0823**	0.2005		**0.0081**
Kal A	0.4168	0.4761	**0.0052**	**0.0418**	

P-values obtained by the Mann–Whitney *U* test between the d values obtained by Eq. (3) for the different materials (below the diagonal) and between the DR slopes in the M_t100%_ − M_t65%_ range for the different materials (above the diagonal). Values below the threshold of statistical significance (0.10) are indicated in bold.

As previously mentioned, d values are always very close to 1 (non-Fickian diffusion) with differences between materials (from 0.969 ± 0.015 for Senofilcon A to 0.990 ± 0.007 for Omafilcon A). The DR slope (taken from Fig. 1) varies from a positive value (1.162E−4 s^−1^ for Senofilcon A corresponding to the P2 mentioned in the literature^1^) to a negative value (− 2.109E−4 s^−1^ for Kalifilcon A corresponding to the P1 mentioned in the literature^1^). In other words, the different positions of the range of interest, i.e., from M_t100%_ to M_t65%_, with respect to the minimum DR (namely the sign of the DR slope which can be either positive or negative) always correspond to a strong non-Fickian diffusion, but allow to compare different materials similarly as the dehydration kinetics coefficient d.

The present study has a limitation in that it does not explore the in-vivo properties of the lenses. This is recognized as a constraint, especially given the existing literature discussing the questionable correlation between in-vivo and in-vitro dehydration results, as evidenced in several studies^21,28,33–35^. Supplementing these data with additional investigations becomes essential to potentially delineate a framework for predicting in-vivo performance. Nonetheless, the present work contributes to the discussion by proposing a method to deduce a parameter describing the dehydration characteristics of different lens materials and acknowledging the need for a comprehensive understanding that integrates various factors influencing in-vivo performance. Indeed, the studies present in the literature collectively highlight the diverse perspectives on this correlation between in-vitro and in-vivo CL performance. For example, Pritchard and Fonn^33^ focused on dehydration, lens movement, and dryness ratings of Hy CLs. Their investigation revealed a weak yet significant correlation between in-vitro dehydration and eye dryness in one of the hydrogel materials studied. This aligns with the notion that the relationship between in-vitro and in-vivo performance is nuanced and may vary across different lens materials.

While Pritchard and Fonn suggested a possible weak correlation^33^, McConville et al. emphasize the limitations of predicting on-eye dehydration based solely on in-vitro data^28^. Their findings underscore the challenges in extrapolating from in-vitro experiments to real-world scenarios, aligning with the broader theme that in-vivo performance is complex and may not be solely determined by in-vitro dehydration properties. The complexity of this relationship is further underscored by the multifaceted nature of lens aging^35^ and the need to consider environmental factors in in-vitro assessments^21^.

## Conclusions

In-vitro dehydration of CLs can be described from different perspectives, including the process of diffusion of water inside the material through the Fick’s model and the corresponding water kinetics coefficient (d) as an indicator to characterize and compare different materials. This coefficient, derived by fitting the curve (VD) which describes the cumulative amount of water loss as a function of time with respect to the total amount of water at the EWC, is strongly correlated with the slope obtained by a linear fitting of the DR curve.

The present study extends this analysis to compare the in-vitro dehydration performances of five CLs, including the recently developed Kalifilcon A, under controlled environmental conditions mirroring typical exposure scenarios (34 °C and 60% relative humidity). The comparison reveals that all materials dehydrate following a non-Fickian model, but statistically significant differences were found between the materials both in terms of dehydration kinetics coefficient and in terms of DR slope. Etafilcon A showed a similar behavior to Omafilcon A, being both Hy CLs, but different from Senofilcon A and Delefilcon A, both SHy CLs. However, Kalifilcon A SiHy CL displayed a hydrogel-like dehydration kinetics.

Despite the debate in establishing a link between in-vitro assessments and in-vivo performance, the current work introduces a method to deduce parameters describing material dehydration characteristics from in-vitro curves. While the analyses focus on specific environmental conditions, it prompts future considerations for replication under diverse conditions and on worn lenses. This approach aligns with the understanding that in-vivo performances may not be solely determined by in-vitro dehydration measurements, emphasizing the need for a broader framework to predict real-world lens performance.

## Data Availability

Data available on request from the authors. Please contact the corresponding author (Erika Ponzini, erika.ponzini@unimib.it) to request the data from this study.
